# Identification of *In Vivo* Metabolites of Dictamnine in Mice Using HPLC-LTQ-Orbitrap Mass Spectrometry

**DOI:** 10.1155/2018/3567647

**Published:** 2018-12-18

**Authors:** Yudong Fu, Yujie Deng, Qing Yu, Xuxia Meng, Dabo Wang, Pei Wang, Ping Wang

**Affiliations:** ^1^Department of Endocrinology, The Affiliated Hospital of Qingdao University, 16 Jiangsu Road, Qingdao 266071, China; ^2^Department of Ophthalmology, The Affiliated Hospital of Qingdao University, 16 Jiangsu Road, Qingdao 266071, China; ^3^School of Pharmacy, Shenyang Pharmaceutical University, Shenyang 110016, China

## Abstract

Dictamnine (4-methoxyfuro[2,3-b]quinolone, DIC), a common furoquinoline alkaloid in the family of Rutaceae, showed diverse biological activities. To investigate the *in vivo* metabolic pathways of DIC, metabolism of DIC in mice was studied using a high-performance liquid chromatography coupled to electrospray ionization of hybrid linear trap quadrupole orbitrap (HPLC-LTQ-Orbitrap) mass spectrometer. Nine metabolites were identified in the DIC-treated mouse urine, plasma, and fecal samples, of which two were identified as new metabolites. The major metabolic pathways of DIC in animal and human liver microsomes were confirmed in the present study, including o-demethylation, monohydroxylation, *N*-oxidation, and 2,3-olefinic epoxidation pathways. For the first time, a mono-acetylcysteine conjugate of DIC (M9) was detected from DIC-treated mouse urine and plasma samples, and 4-methoxy-2-oxo-1,2-dihydroquinoline-3-carboxylic acid (M10) and 2-(2,8-dihydroxy-4-methoxyquinolin-3-yl)acetaldehyde (M11) were identified as new metabolites of DIC; furthermore, using an *in vitro* human fecal incubation model, furo[2,3-b]quinolin-4-ol (M1) was verified to be a microbial demethylated metabolite of DIC. Collectively, the present study provided new information on the *in vivo* metabolic fate of DIC.

## 1. Introduction

Dictamnine (4-methoxyfuro[2,3-b]quinolone, DIC), a common furoquinoline alkaloid in the family of Rutaceae, showed diverse biological activities, such as antifungal, antibacterial, vascular-relaxing, antiplatelet aggregation, and antihypertension activities [1–7]. Moreover, DIC exhibits potent cytotoxic activity against human cervix, colon, breast, and lung cancer cell lines, and the main mechanism is by inhibiting cell proliferation and promoting cell apoptosis [8, 9]. Recently, Liang et al. reported that DIC as an anticancer agent arrests cells in the S phase of the cell cycle and induces human lung adenocarcinoma A549 cell apoptosis via the mitochondria-mediated caspase-independent pathway [10]. Furthermore, novel synthetic and semisynthetic derivatives of DIC, as potential antitumor agents, have gained increasing attention in medicinal research [11].

Our previous studies have demonstrated that DIC is a promising drug candidate with favorable oral pharmacokinetic profiles [12, 13]. Very recently, using *in vitro* microsomal incubation systems, we systematically studied the metabolites of DIC in human and animal liver microsomal incubation systems [14], and we found that the metabolic fate of DIC in human liver microsomes was about the same as that in mouse liver microsomes, while with some differences compared with that in rat, dog, and monkey liver microsomes. We elucidated that the o-demethylation, monohydroxylation, *N*-oxidation, and 2,3-olefinic epoxidation pathways are the major metabolic routes of DIC [14]. In a separate study [15], a *N*-acetylcysteine (NAC) conjugate which was derived from the reactive DIC metabolite, 2,3-epoxide DIC, was detected in the rat liver microsomal incubation system, as well as in DIC-treated rat urine samples.

It is also remarkable to note that there are no reports about the *in vivo* metabolism study of DIC in either humans or mice. Choosing the most relevant animal species to conduct studies is essential for researchers to extrapolate results from studies performed in animals onto human [16–19]. In the preset study, based on the previous results [14, 15], the *in vivo* metabolism study of DIC was performed on mice. A powerful hybrid linear quadrupole ion trap orbitrap (LTQ-Orbitrap) mass spectrometer equipped with a high-performance liquid chromatography (HPLC) system was used for the structural elucidation of the metabolites.

## 2. Materials and Methods

### 2.1. Reagents

DIC, purity higher than 98%, was purchased from National Institute for the Control of Pharmaceutical and Biological Products (Beijing, China). HPLC-MS-graded acetonitrile (ACN), methanol (MeOH), and formic acid were obtained from Fisher Scientific (Pittsburgh, PA, USA). Purified water was obtained from a Milli-Q system (Millipore, Bedford, USA).

### 2.2. Treatment of Mice and Sample Collections

Experiments with mice were carried out with male Kunming mice (25 ± 2 g) which were obtained from the Experimental Animal Center of Qingdao University (Qingdao, China). All mice were housed 5 per cage with the 12 h light/dark cycle at an ambient temperature of 20 ± 2°C with a relative humidity of 50 ± 10%. Mice were allowed to acclimate for at least 1 week prior to the start of the experiment. Mice were fed with AIN-93 semipurified diet and were allowed *ad libitum* food and water throughout the study. Metabolic cages were used for the collection of twenty-four-hour mouse urine and fecal samples. In brief, DIC in DMSO or DMSO only were gavaged to mice individually, and mouse feces and urine were collected using metabolic cages (5 mice per cage) for 24 h after administration of DIC (the treated group, *n*=5, 5 mg/kg body weight) or vehicle (the control group, *n*=5). The mouse blood sample was collected from anesthetized mice by a cardiac puncture at 2 h after administration of vehicle or DIC, and the whole blood sample was centrifugated at 8,000 × g for 10 minutes at 4°C to isolate plasma. Before analysis, all samples were stored at −80°C.

### 2.3. Fecal, Urinary, and Plasmatic Sample Preparation

For acquisition of the metabolic profiles, six pieces of each fecal sample (control and treated) were chosen and homogenized with 1 mL of MeOH/H_2_O/acetic acid (90/10/0.1, v/v/v) solution for 3 minutes and then centrifuged at 12,000 × g for 10 minutes at 4°C. An aliquot (200 *μ*L) of the supernatant was picked up and diluted for 20 times before analysis. The urine and plasma samples were deconjugated by the enzyme as described previously with slight modifications [20, 21]. In brief, 100 *μ*L from each sample (control and treated) was incubated with *β*-glucuronidase (250 U) and sulfatase (3 U) for 90 min in a water bath at 37°C. The reaction was quenched by adding an equal volume of MeOH containing 1% acetic acid and centrifuged at 12,000 × g for 10 minutes at 4°C. Aliquots of the supernatant were stored at −20°C before analysis.

### 2.4. *In Vitro* Fecal Batch Fermentation Experiments

Three healthy volunteers (mean age, 32.5-years-old; mean weight, 69.5 kg; nonsmokers) were involved in this study, all of them were antibiotic-free for over 6 months before the study begins, and they had not taken polyphenol-rich foods for over two days before the stool sample collection. Stool samples were collected and transported immediately to the anaerobic condition and were processed within 2 h. Under anaerobic conditions, 50 g of the fresh collected stool sample was homogenized in a sterilized stomacher bag with 100 mL of water containing 0.05% peptone. The particulate materials were removed by centrifugation at 3000 rpm for about 2.5 min, and then the supernatant of the stool slurry was mixed with 35% presterile glycerol and stored at −80°C before use. For the *in vitro* fermentation experiment, a pooled human stool slurry was made by direct mixing of an equal volume of the stool slurry from each subject. The fermentation basal medium contains about 4% of Tween 80, and 2% of peptone was freshly prepared and autoclaved before study. Five *μ*L of DIC solution (5 mg/mL in DMSO), 5 mL of fermentation medium, and 0.5 mL of pooled human stool slurry were mixed and then aliquoted into 3 sets of samples (∼1.5 mL each) to represent 0, 12, and 24 h time points. Under anaerobic conditions, samples were fermented at 37°C and harvested at the corresponding time intervals. Once harvested, 450 *μ*L of the supernatant was picked up and quenched by adding 900 *μ*L of acetonitrile (0.2% acetic acid). Samples were centrifuged at 12,000 × g for 10 min, and then aliquots (200 *μ*L) of the supernatants were transferred to HPLC vials for analysis.

### 2.5. HPLC-LTQ-Orbitrap Conditions

HPLC was performed on a HPLC system comprised degassers, a quaternary pump, a column compartment, and an autosampler from Thermo-Finnigan (Thermo Electron Co., USA). A Phenomenex Gemini C18 column (3.0 mm × 150 mm; 5 *μ*m; Torrance, CA, USA) was used for separation with column temperature set at 35°C. The HPLC conditions were the same as we described before [14]. In brief, the composition of the mobile phase was 5% water with 95% methanol containing 0.1% formic acid as mobile phase A and 95% water with 5% methanol containing 0.1% formic acid as mobile phase B. Gradient elution program was initiated with 10% B for 5 min, then increased to 25% from 5 to 10 min, to 60% from 10 to 30 min, to 100% from 30 to 40 min, and then held constant for 5 min. The column was equilibrated with 10% B for 5 min between each run. The flow rate was set at 0.3 mL/min, and 10 *µ*L was injected for each sample.

A Finnigan LTQ-Orbitrap XL mass spectrometer coupled with an electrospray ionization (ESI) interface (Thermo Electron, Bremen, Germany) was used for the mass spectra collection. Nitrogen was used as a sheath and auxiliary gas with 30 and 10 arbitrary units, respectively, and helium was applied as the collision gas. The tube lens and capillary voltage were set at 65 V and 250 V, respectively. The mass spectrometer was set to repeat a cycle consisting of a full MS scan followed by an MS/MS scan. Mass spectra were collected from *m/z* 50 to 500 amu in the positive ionization mode. Collision-induced dissociation (CID) energy was set at 35 for MS^n^ analysis with an isolation width set at 1.5 amu and activation time set at 30 ms, respectively. Data acquisition and analysis were performed using Xcalibur software (2.2 version, Thermo Electron, San Jose, CA, USA).

## 3. Results

### 3.1. Metabolic Fate of DIC in Mice

**Figure 1:**
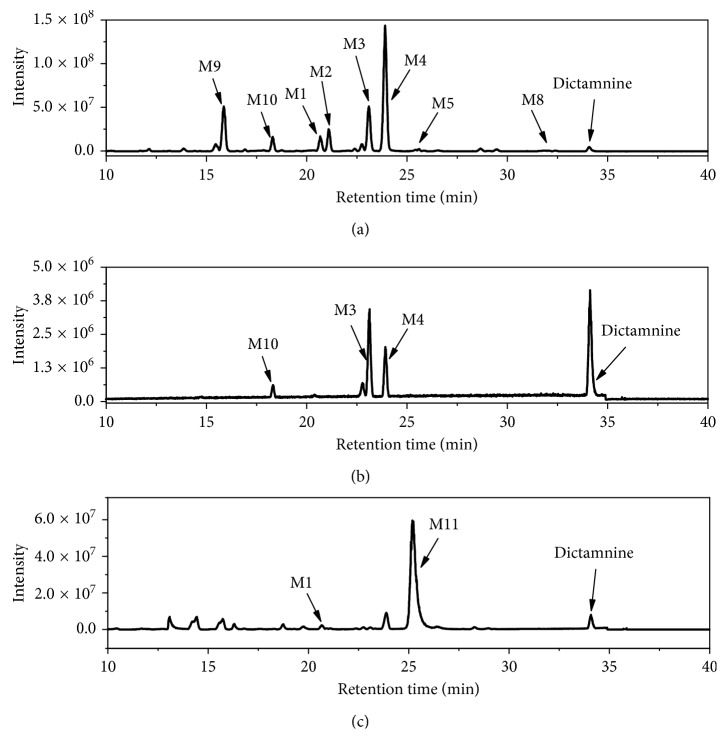
Extracted ion chromatograms of dictamnine and its metabolites in mouse urine (a), plasma (b), and feces (c).

In present study, HPLC-LTQ-Obitrap/MS was used to explore the major metabolites of DIC in samples. Extracted ion chromatograms (EIC) of the metabolites detected from the DIC-treated mouse fecal, plasmatic, and urinary samples are shown in Figure 1. Compared to the samples collected from vehicle-treated mice, nine major metabolites were observed. M1–M5 and M8 were numbered according to our previous study, and new identified metabolites (M9–M11) were numbered sequentially to their chromatographic retention times. By comparing the LC/MS^n^ data and retention times to our previously microsomal studies and other literatures [14, 22, 23], six metabolites were unequivocally identified as furo[2,3-b]quinolin-4-ol (M1), 3-(1,2-dihydroxyethyl)-4-methoxyquinolin-2(1H)-one (M2), 3-(2-hydroxyethyl)-4-methoxyquinolin-2(1H)-one (M3), 4-methoxyfuro[2,3-b]quinolin-6-ol (M4), robustine (M5), and 4-methoxy-furo[2,3-b]quinoline-9-oxide (M8). The structures of M9–M11 were tentatively elucidated by interpretation of their accurate molecular weight and characteristic ions collected from LC-LTQ-Obitrap/MS.

### 3.2. Identification of M9 (NAC-DIC Conjugate)

**Figure 2:**
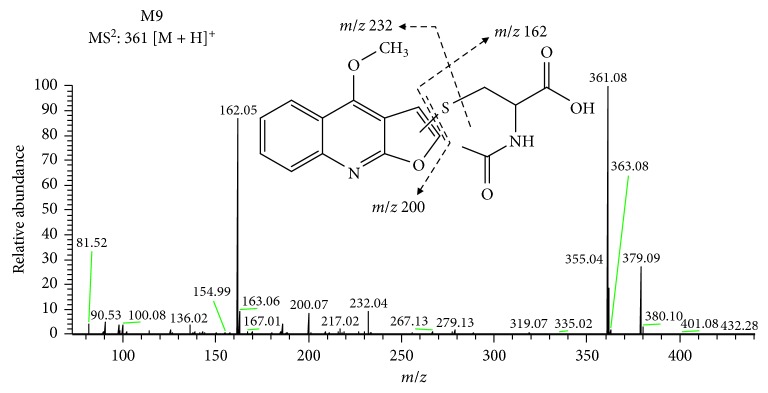
The proposed fragmental pathway and the MS^2^ bar graph of M9.

M9 was observed with a protonated molecular weight of *m/z* at 361.0854 [M + H]^+^, and the molecular formula was calculated as C_17_H_17_O_5_N_2_S, which was 161 amu higher than [M + H]^+^ of DIC, indicating a NAC incorporation in DIC. MS^2^ spectra of M9 (Figure 2) shows the characteristic product ions at *m/z* 232.0431 [M + H − C_5_H_8_NO_3_]^+^, 200.0708 [M + H − C_5_H_8_NO_3_]^+^, and 162.0219 [M + H − C_5_H_8_NO_3_S]^+^, suggesting this metabolite was the mono-NAC adduct of DIC. The MS^2^ fragment ion of *m/z* 200.0708 corresponding to the loss of one NAC molecule and the tandem mass spectra of this fragment ion (MS^3^: 200.0708/361.0854) were identical to those of DIC, indicating that the structure of DIC remains unchanged [14, 15]. This result suggests the NAC was conjugated to the furan ring.

### 3.3. Identification of Metabolites M10 and M11

**Figure 3:**
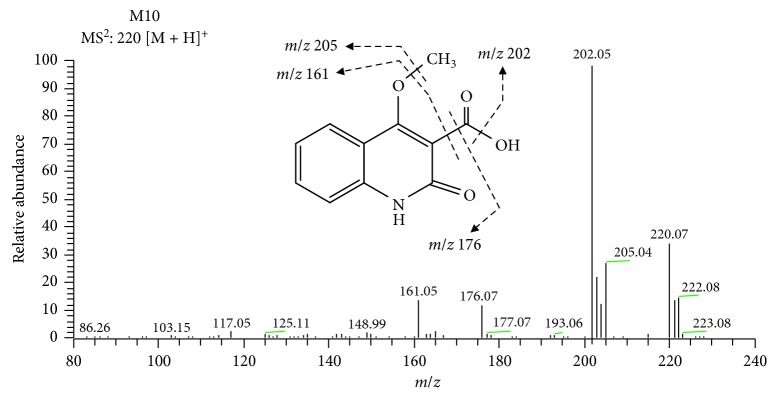
The proposed fragmental pathway and the MS^2^ bar graph of M10.

Another two metabolites, M10 and M11, were tentatively identified based on their accurate mass weights and fragmentation patterns. M10 was identified with a protonated molecular weight of *m/z* at 220.0609 [M + H]^+^, and its molecular formula was calculated as C_11_H_10_NO_4_, which was 20 amu higher than [M + H]^+^ of DIC. MS^n^ spectra of M10 (Figure 3) generated the characteristic fragment ions at *m/z* 205.0371 [M + H − CH_3_]^+^, *m/z* 202.0609 [M + H − H_2_O]^+^, *m/z* 176.0712 [M + H − CO_2_]^+^, and *m/z* 161.0475 [M + H − CO_2_ − CH_3_]^+^. In summary, M10 was tentatively identified as dictamnic acid.

**Figure 4:**
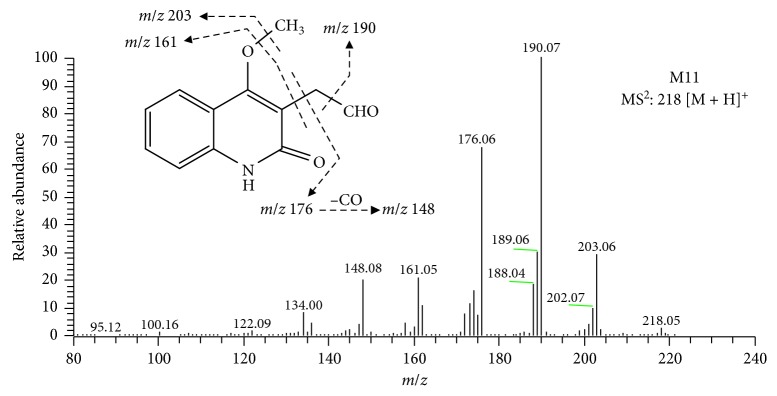
The proposed fragmental pathway and the MS^2^ bar graph of M11.

M11 exhibits a molecular weight of 217 amu as determined by the mass ion at *m/z* 218.0815 [M + H]^+^, which was 18 amu higher than [M + H]^+^ of DIC. The formula was calculated to be C_12_H_12_NO_3_. MS^2^ spectra (Figure 4) of M11 generated the characteristic product ions at *m/z* 203.0581 [M + H − CH_3_]^+^, *m/z* 190.0867 [M + H − CO]^+^, *m/z* 176.0710 [M + H − C_2_H_2_O]^+^, *m/z* 161.0475 [M + H − C_2_H_2_O − CH_3_]^+^, and *m/z* 148.0759 [M + H − C_2_H_2_O − CO]^+^, supporting that M11 is 2-(2-hydroxy-4-methoxyquinolin-3-yl)acetaldehyde.

### 3.4. Metabolism of DIC by Human Fecal Gut Microbiota

**Figure 5:**
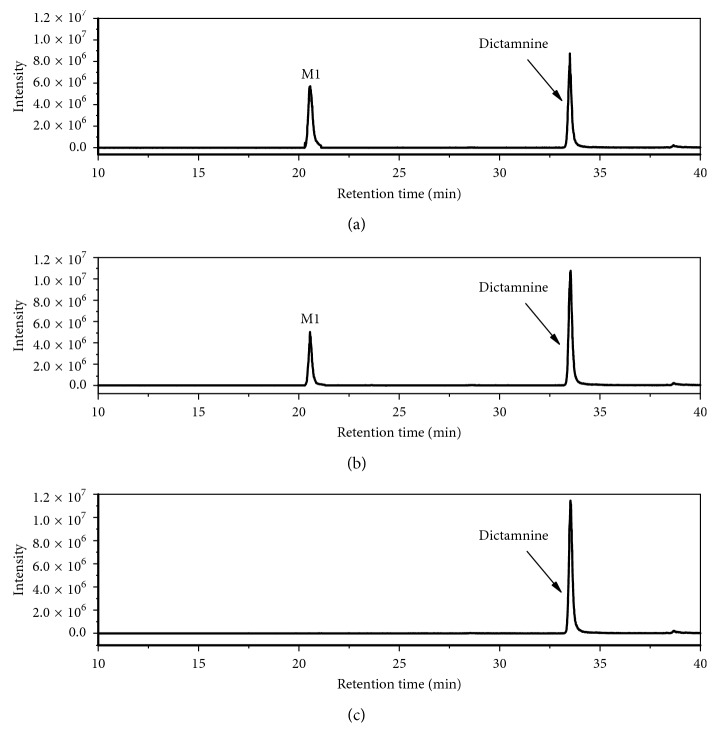
Representative extracted ion chromatograms of microbial metabolites of dictamnine after incubating with pooled human fecal slurry. (a) 24 h; (b) 12 h; (c) control.

To study the impact of gut microbiota for the *in vivo* metabolism of DIC, DIC was incubated with the pooled human stool slurries obtained from three healthy individuals. Samples were harvested as a function of time and analyzed by LC-MS for the elucidation of the microbial-derived metabolites.  Figure 5 shows a representative extracted ion chromatograms of DIC metabolites after incubating with the pooled human stool slurry. DIC was digested progressively with increasing time. Compared with the control sample (collected from 0 h time point), a new peak, retention time at 20.6 min, was detected from the sample collected at the time point of 12 h, and the peak area of this peak increased with increasing time. This peak was identified as furo[2,3-b]quinolin-4-ol (M1) by comparing its retention time and tandem mass spectra with those collected from DIC-treated mouse urine and fecal samples. Microbial pathway of o-demethylation plays a crucial role in the transformation of polyphenols and other drugs after absorption; furthermore, o-demethylation influences the antioxidative properties of those phenolic compounds by liberating free hydroxyl groups, and recent studies have shown that the gut microbiota is able to demethylate peonidin, vanilic acid, anthocyanins, and their phenolic degradation products [24, 25].

## 4. Concluding Remarks

**Figure 6:**
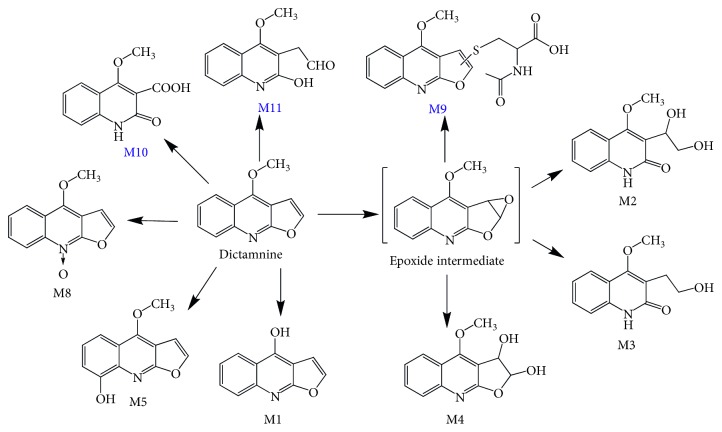
Chemical structures of the nine identified metabolites and their potential metabolic pathways in mice.

In summary, our present study showed that DIC, a common furoquinoline alkaloid belongs to the family of Rutaceae, can be extensively metabolized in mice. In total, nine metabolites were identified in mouse urinary, plasmatic, and fecal samples, and two of those (M10 and M11) were identified for the first time. The metabolic profile of DIC in the mouse was finally elucidated (Figure 6).

Additionally, our present study showed that the gut microbiota intermediates the o-demethylation of DIC, which results in the demethylation metabolite of DIC, M1. Finally, the metabolic pathway of DIC in mice was proposed, and these data are significant for further researches of DIC and allows intelligent assessment of preclinical toxicology and/or *in vivo* bioactivity studies performed on mice.

## Data Availability

The data used to support the findings of this study are available from the corresponding author upon request.
