# Corrigendum to “Identification of In Vivo Metabolites of Dictamnine in Mice Using HPLC-LTQ-Orbitrap Mass Spectrometry”

**DOI:** 10.1155/2019/5451838

**Published:** 2019-04-02

**Authors:** Yudong Fu, Yujie Deng, Qing Yu, Xuxia Meng, Dabo Wang, Pei Wang, Ping Wang

**Affiliations:** ^1^Department of Ophthalmology, The Affiliated Hospital of Qingdao University, 16 Jiangsu Road, Qingdao 266071, China; ^2^Department of Endocrinology, The Affiliated Hospital of Qingdao University, 16 Jiangsu Road, Qingdao 266071, China; ^3^School of Pharmacy, Shenyang Pharmaceutical University, Shenyang 110016, China

In the article titled “Identification of In Vivo Metabolites of Dictamnine in Mice Using HPLC-LTQ-Orbitrap Mass Spectrometry” [1], the first and second affiliations were reversed. The revised affiliations are shown above.
